# Association of hypertension with coronary artery disease onset in the Lebanese population

**DOI:** 10.1186/2193-1801-3-533

**Published:** 2014-09-16

**Authors:** Aline Milane, Jad Abdallah, Roy Kanbar, Georges Khazen, Michella Ghassibe-Sabbagh, Angelique K Salloum, Sonia Youhanna, Aline Saad, Hamid El Bayeh, Elie Chammas, Daniel E Platt, Jörg Hager, Dominique Gauguier, Pierre Zalloua, Antoine Abchee, FGENTCARD Consortium

**Affiliations:** Lebanese American University, School of Pharmacy, Byblos 36, Lebanon; School of Arts and Sciences, Lebanese American University, Byblos 36, Lebanon; School of Medicine, Lebanese American University, Beirut, 1102 2801 Lebanon; Bioinformatics and Pattern Discovery, IBM T. J. Watson Research Centre, Yorktown Hgts, NY 10598 USA; CEA-Genomics Institute, Centre National de Génotypage, Evry, 91057 France; The Wellcome Trust Centre for Human Genetics, University of Oxford, Roosevelt Drive, Headington, Oxford, OX3 7BN UK; INSERM UMRS872, Centre de Recherche des Cordeliers, 15 Rue de l’école de Médecine, Paris, 75006 France; Harvard School of Public Health, Boston, MA 02215 USA; Department of Internal Medicine, American University of Beirut, Beirut, Lebanon

**Keywords:** Hypertension, Coronary artery disease (CAD), Risk factors, Antihypertensive drugs, Therapeutic lifestyle changes, Lebanese population

## Abstract

The onset of coronary artery disease (CAD) is influenced by cardiovascular risk factors that often occur in clusters and may build on one another. The objective of this study is to examine the relationship between hypertension and CAD age of onset in the Lebanese population.

This retrospective analysis was performed on data extracted from Lebanese patients (n = 3,753). Logistic regression examined the association of hypertension with the age at CAD diagnosis after controlling for other traditional risk factors. The effect of antihypertensive drugs and lifestyle changes on the onset of CAD was also investigated.

Results showed that hypertension is associated with late onset CAD (OR=0.656, 95% CI=0.504-0.853, p=0.001). Use of antihypertensive drugs showed a similar association with delayed CAD onset. When comparing age of onset in CAD patients with traditional risk factors such as hypertension, diabetes, hyperlipidemia, obesity, smoking and family history of CAD, the age of onset was significantly higher for patients with hypertension compared to those with any of the other risk factors studied (p < 0.001).

In conclusion, hypertension and its treatment are associated with late coronary atherosclerotic manifestations in Lebanese population. This observation is currently under investigation to clarify its genetic and/or environmental mechanisms.

## Introduction

Cardiovascular diseases have become a very prevalent public health problem in both developed and developing countries. In Lebanon, coronary artery disease (CAD) is believed to be one of the leading causes of death (Sibai et al. 2001). CAD risk factors were first described in studies in the mid-twentieth century (Dawber et al. 1959; Arnaout et al. 2011). Hypertension, one of the most traditional risk factors, has been consistently correlated with increased probability of developing CAD in various populations (Dawber et al. 1959; Lewington et al. 2002; Lakka et al. 1999; Collins et al. 1990; MacMahon et al. 1990). The epidemiological studies are supported by experimental evidence postulating that hypertension predisposes to atherosclerosis through a shared synergistic mechanism involving inflammation and oxidative stress in the arterial wall (Li JJChen 2005; O'Keefe et al. 2009).

The association of hypertension with CAD manifestations onset has not been thoroughly investigated in Middle Eastern populations. A limited number of studies showed that there is a significant association between hypertension and acute myocardial infarction (MI) in older patients (Sengul et al. 2011; Zuhdi et al. 2013). One study however described hypertension as one of the most frequent risk factors for premature CAD (Sadeghi et al. 2013).

It is conceivable that the effect of hypertension on CAD disease onset may be modulated by various environmental and genetic factors. However, it is widely accepted that strategies adopted to lower blood pressure play a protective role by delaying atherosclerotic lesion formation (Simon ALevenson 2002; Tropeano et al. 2011).

The present study was designed to investigate the association between hypertension and CAD age of onset in Lebanese patients who were recruited as part of a multi-center cross-sectional study for the FGENTCARD project. Furthermore, the association of pharmacological and non-pharmacological anti-hypertensive strategies was examined.

## Methods

### Study subjects and collection of data

A total of 5,347 Lebanese patients undergoing cardiac catheterization were sequentially enrolled for the FGENTCARD study (Youhanna et al. 2010) as part of a multi-center cross-sectional study conducted at the Lebanese American University, the Rafic Hariri University Hospital and the “Centre Hospitalier du Nord” Lebanon, between May 2007 and June 2010. The Institutional Review Board at the Lebanese American University approved the study protocol and all subjects gave informed consent before their enrollment. Catheterization was performed by Judkins’ technique.

Among the 5,347 enrolled subjects, 1,594 had no or minor observable lesions in all coronary arteries and 3,753 patients presented coronary lesions that are classified as mild (≤50% stenosis in at least one vessel) or severe (>50% stenosis in one or more of the coronary arteries). The age of CAD onset was defined as the age upon first diagnosis of CAD by catheterization. Since gender is known to influence disease onset (Abchee et al. 2006), male patients in our study population were categorized as having early onset CAD if diagnosed at an age younger than or equal to 45 (≤45), while female patients were categorized as having early onset CAD when diagnosed at an age younger than or equal to 55 (≤55). Accordingly, the 3,753 CAD patients were divided into two groups: early onset CAD (n = 415) and late onset CAD (n = 3,338) depending on their age at diagnosis.

A questionnaire specifically developed to measure the impact of CAD risk factors and family history of CAD (FxCAD) was duly filled and signed by each participant. Diabetes, hypertension and hyperlipidemia were noted when the condition was reported by an ascertained physician. Body Mass Index (BMI) was calculated according to standard measurements. Smokers were defined as subjects who smoked cigarettes before or at the time of enrollment for the study. Physical activity level was determined according to the daily number of exercising hours (inactive, moderate activity, and regular exercise). Annotations were coded from medical charts for additional data such as laboratory tests, nutritional diet (normal or low-salt), prescribed medications, and presence of other diseases and conditions (Youhanna et al. 2010).

### Regression models and statistical analysis

A binomial logistic regression model was used to estimate the odds of having an early onset (1) versus late onset CAD (0) using the covariates: hypertension, lifestyle changes (being on a low-salt diet, and reporting moderate or regular physical activity) and the use of one or more anti-hypertensive drug. Odds ratios were adjusted after controlling for cofounding variables (smoking, obesity, diabetes, hyperlipidemia, and FxCAD) as well as for use of other drugs (metabolic, anticoagulants and anti-arrhythmics: data not shown).

In addition, we compared CAD age of onset between different patients groups based on the presence or absence of hypertension combined with one or more CAD traditional risk factors. The age of onset of CAD among these different groups was compared using an Analysis of Variance (ANOVA) test and the Tukey Honest Significant Differences (TukeyHSD) test was performed for paired multiple comparisons.

The R statistical software (version 2.14) was used for the analysis and the glm (generalized linear models) function from the “stats” package was used for building the logistic regression model. A p value of 0.05 indicated statistical significance. The average area under the curve (AUC) was computed for the logistic regression model.

## Results

The study population consisted of 72% males and 28% females. Mean CAD age of onset was 61.0 ± 11.0 years (early CAD onset average age: 44.6 ± 6.0 years, late CAD onset average age: 62.9 ± 9.7 years). 11.1% of patients had early CAD onset. Table 1 describes the distribution of patients according to CAD associated risk factors. The dominant majority of CAD cases were associated with at least one traditional risk factor with 63.9% reported having high blood pressure. CAD patients with no risk factor represented only 1.7% of the total population.

**Table 1:** **Descriptive statistics of patients population (n (% by column))**

		Early CAD	Late CAD	Total
		n = 415	n = 3338	n = 3753
**Age**		47.0 ± 6.7	64.7 ± 9.5	62.4 ± 10.9
**Age of onset**		44.6 ± 6.0	62.9 ± 9.7	61.0 ± 11.0
**Gender**	**Male**	218 (52.5)	2486(74.5)	2704 (72)
	**Female**	197 (47.5)	852 (25.5)	1049 (28)
**Diabetes**	**No**	288 (69.4)	2175 (65.2)	2463 (65.6)
	**Yes**	127 (30.6)	1163 (34.8)	1290 (34.4)
**Hypertension**	**No**	192 (46.3)	1163 (34.8)	1355 (36.1)
	**Yes**	223 (53.7)	2175 (65.2)	2398 (63.9)
**Hyperlipidemia**	**No**	182 (43.9)	1592 (47.7)	1774 (47.3)
	**Yes**	233 (56.1)	1746 (52.3)	1979 (52.7)
**Obesity**	**No**	230 (55.4)	2050 (61.4)	2280 (60.8)
	**Yes**	185 (44.6)	1288 (38.6)	1473 (39.2)
**Smoking**	**No**	111 (26.7)	1185 (35.5)	1296 (34.5)
	**Yes**	304 (73.3)	2153 (64.5)	2457 (65.5)
**FxCAD**	**No**	100 (24.1)	1339 (40.1)	1439 (38.3)
	**Yes**	315 (75.9)	1999 (59.9)	2314 (61.7)

### Association of risk factors with early vs. late CAD onset

A logistic regression model (Table 2) was built to assess the impact of various risk factors on disease onset (early versus late CAD). This model showed that smoking (OR = 1.864, p < 0.001), obesity (OR = 1.237, p < 0.05) and FxCAD (OR = 1.968, p < 0.001) were positively associated with early onset of CAD, contrary to hyperlipidemia and diabetes which did not show significant association with early disease onset (p > 0.05).

**Table 2:** **Adjusted odds ratios predicting early CAD as outcome variable**

	Early onset CAD odds ratio	95% confidence interval (CI)	p value
**Hypertension**	0.656	0.504-0.853	0.0016
**Anti-** **hypertensive drugs**	0.635	0.483-0.836	0.0011
**Lifestyle changes**	1.804	0.985-3.647	0.0746
**Smoking**	1.864	1.466-2.387	<0.001
**Obesity**	1.273	1.023-1.583	0.0300
**Diabetes**	0.803	0.625-1.025	0.0812
**Hyperlipidemia**	1.132	0.909-1.411	0.2695
**FxCAD**	1.968	1.551-2.516	<0.001

Hypertension and the use of anti-hypertensive drugs were separately significantly associated with late onset of CAD (OR = 0.656, p = 0.001; OR = 0.635, p = 0.001, respectively). Lifestyle changes were not significantly associated with early CAD onset (OR = 1.804, p > 0.05). The AUC of the logistic regression model was found to be 0.684.

Most CAD patients (69.8%, n = 2935) were treated with antihypertensive drug(s); among them, 43% (n = 1263) received a single drug which was for most cases (52.81%, n = 667) a β-blocker. The following were the antihypertensive drugs reported by our study patients: Calcium Channel Blockers (CCBs), β-blockers, Angiotensin Conversion Enzyme Inhibitors (ACEIs), Angiotensin II Antagonists (AIIAs) and diuretics.

### Comparison of age of CAD onset between risk factors groups

**Figure 1:**
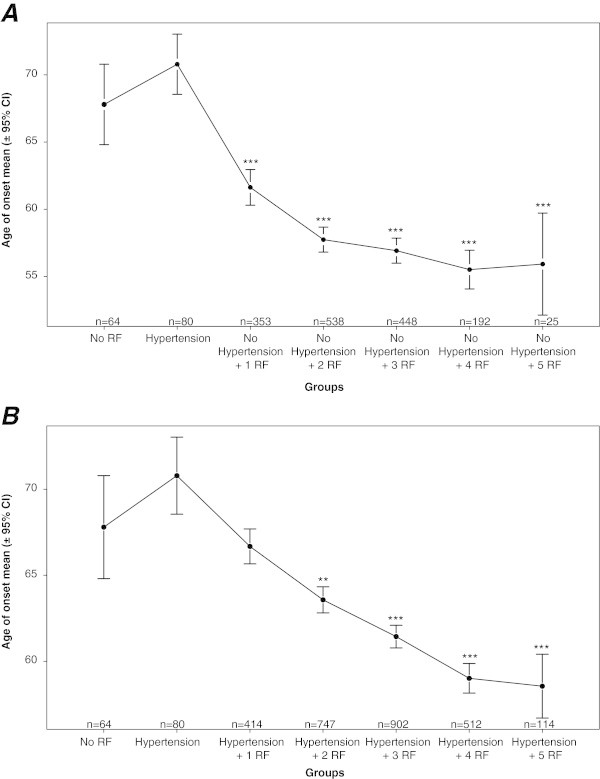
**Comparison between the mean ages of onset (±95% CI) of CAD among several groups of CAD patients sorted according to the associated risk factors (RF).** CAD patients within the group “hypertension” had only hypertension as RF. The group “no RF” had no observed or documented RF. **A**. CAD age of onset in non-hypertensive patients with cumulative RF was compared between the various CAD groups (ANOVA) and then each group to the “hypertension” group (Tukey HSD). CAD patients within group “no hypertension + 1 RF” had any of the following RF: smoking, obesity, diabetes, hyperlipidemia and FxCAD, but did not have hypertension. In subsequent groups, CAD patients had any two, three, four or all five RF, but no hypertension. (*p < 0.05, **p < 0.01, ***p < 0.001 *vs*. hypertension group). **B**. CAD age of onset in hypertensive patients with cumulative RF was compared between the various CAD groups and then each group to the “hypertension” group. CAD patients in group “hypertension + 1 RF” had hypertension in addition to anyone of the other previously mentioned RF. In subsequent groups, CAD patients with hypertension had any two, three, four or all five additional RF. HSD (*p < 0.05, **p < 0.01, ***p < 0.001 *vs*. hypertension group).

**Figure 2:**
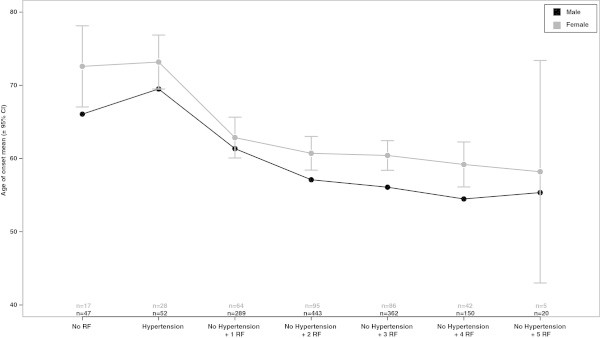
**Mean age (±95% CI) distribution of CAD age of onset according to gender (black for men and grey for women).** CAD patients within the group “hypertension” had only hypertension as RF. The group “no RF” had no observed or documented RF. CAD age of onset in non-hypertensive patients with cumulative RF was compared between the groups (ANOVA) and then each group to the “hypertension” group (Tukey HSD). CAD patients within group “no hypertension + 1 RF” had any of the following RF: smoking, obesity, diabetes, hyperlipidemia and FxCAD, but did not have hypertension. In subsequent groups, CAD patients had any two, three, four or all five RF, but no hypertension.

When comparing CAD age of onset among patients with various risk factors, results showed that there is a significant effect of risk factors on the age of onset of CAD (ANOVA test, p < 0.001). In addition, paired comparisons using the TukeyHSD test showed that the age of onset of CAD in patients with hypertension was significantly higher than the age of onset of CAD in patients with any one or more of the other risk factors (Figure 1A). The difference in CAD age of onset persisted when patients were subdivided according to gender (Figure 2). Furthermore, CAD age of onset decreased significantly when two or more risk factors were considered in addition to hypertension (Figure 1B).

## Discussion

The major finding of this analysis is that hypertension and its treatment were found to be independently associated with CAD onset at a later age.

### Relationship between hypertension and CAD

This study represents a primary analysis of the relationship between hypertension and time of CAD diagnosis in the Lebanese population. Although previous studies conducted confirm the strong relationship between hypertension and ischemic heart disease in general, it is clear that differences exist between populations on how hypertension affects CAD especially that few studies investigated the direct association between presence of hypertension and onset of CAD (Benfante et al. 1989; Chen et al. 1995; Menotti et al. 2004). Hypertension, as well as diabetes, were dominant risk factors in the older CAD group in a large study done on 15,381 CAD patients in Germany (Reibis et al. 2012). In a population of men of Japanese ancestry, hypertension had a similar effect on both early and delayed CAD (Benfante et al. 1989). In a selective population of Canadian patients, the relative risk of high blood pressure for ischemic heart disease, declined with advancing age (Tate et al. 1998). In a study aiming to explore the association of major coronary risk factors with CAD in a sub-population of men in the United States, it was found that risk of coronary death increased with time as a function of blood pressure (Menotti et al. 2004). In the Middle East countries, limited studies investigated the association between high blood pressure and CAD. While most of them found an association between hypertension and late CAD manifestations (Sengul et al. 2011; Zuhdi et al. 2013), one study reported hypertension as one of the most frequent risk factors in premature CAD (Sadeghi et al. 2013). These differences highlight the fact that socioeconomic, environmental and genetic factors play a determining role in the development of cardiovascular diseases (Watson KETopol 2004).

In the Lebanese population, the prevalence of hypertension has been estimated at 23.1% in a representative sample of the general population and it was higher in the less educated and unemployed (Tohme et al. 2005). The pathogenesis of hypertension includes the interaction of genetic and environmental factors including abnormalities of fluid volume regulation, increased vasoconstriction, and remodeling of the vascular wall (decreasing diameter and increasing resistance). The mechanisms by which hypertension would trigger atherosclerosis have not been well elucidated. Dysfunctional vascular endothelium leads to vascular smooth cell growth, which narrows the lumen. Angiotensin II, through activation of the AT-1 receptor, induces generation of inflammatory mediators and reactive oxygen species in the blood vessel wall and as such plays an active role in the inflammation process (MacKenzie 2011; Alexander 1995). These mechanisms are all slow grade processes that take years to develop. This can partially explain our finding that hypertension was associated with late rather than early CAD.

### Anti-hypertensive strategies and CAD

Moreover, the use of anti-hypertensive drugs showed an association with late CAD as outcome variable. This result is expected since most anti-hypertensive drugs, in addition to reducing blood pressure, are known to be beneficial in lowering cardiovascular disease risks in general. The preservation or recovery of endothelial function in hypertensive patients is crucial to inhibit the development of atherosclerosis and the onset of cardiovascular events. β-blockers improve endothelium-dependent vasodilation, decrease peripheral vascular resistance, and were also shown to have some antioxidant effects (Alexander 1995; Balligand 2009). According to a recent analysis of randomized trials that used ultrasound imaging to measure changes in vascular-disease severity over time, long-term β-blocker therapy was associated with a reduction in atheroma volume (Sipahi et al. 2007). In addition, in a large multi-national randomized prospective trial, INVEST, focusing on CAD patients with hypertension, it was found that treatment strategies utilizing a calcium-channel blocker (CCB) or a β-blocker prevent all-cause mortality and non-fatal myocardial infarction (Pepine et al. 2003). Moreover, ACEIs protect the endothelium from oxidative stress by inhibiting the formation of Angiotensin II, a potent inducer of reactive oxygen species production in vascular cells (White 2007). AIIAs activate bradykinin B2 receptor-mediated nitric oxide production. Both ACEIs and AIIAs, have been shown to reduce atherosclerotic lesion formation (Yusuf et al. 2000; Dahlof et al. 2002; Besler et al. 2008) and intima-media thickness (Cuspidi et al. 2009). Finally, diuretics reduce significantly the occurrence of cardiovascular disease (Psaty et al. 2003) and stroke (Papp et al. 2012).

The logistic regression model corrected the odds ratio of hypertension for the effect of both the pharmacological treatment and lifestyle changes. This seems to favor the hypothesis that other population-specific genetic and/or nutritional factors would explain the observed significant association of hypertension with the late CAD onset in our population.

Many studies have documented genetic susceptibilities to CAD and hypertension, however to-date, these results could not confirm the presence of common susceptibility variants for these two diseases (Slavin et al. 2011; Zhu et al. 2010; Ioannidis 2009). The most current Genotypes and Phenotypes database (dbGaP) lists 29 genetic variants associated with hypertension and 69 associated with CAD. None of these genetic variants overlaps, suggesting the need for additional genome-wide association studies that can unravel common genetic pathways between these two clinically related diseases and identify variants that modulate CAD age of onset in hypertensive patients.

### Study limitations

One of the main limitations of the current study is the lack of information on the duration of hypertension and the elapsed time before treatment initiation which are modulating factors for CAD development.

## Conclusions

Our study presents a primary general observation of the association of hypertension and its treatment with CAD development. Although suggestive, these findings need to be substantiated by a prospective study that we are currently conducting that would confirm our results after controlling for duration since hypertension diagnosis as well as initiation of treatment and therapy goals. Such a prospective study would be ideal to investigate the potential genetic and nutritional variants involved in this association.
